# Supplementary material for: Carbon Felts Uniformly Modified with Bismuth Nanoparticles for Efficient Vanadium Redox Flow Batteries
Source: Nanomaterials (Basel). 2024 Dec 23;14(24):2055. doi: 10.3390/nano14242055 (PMC11676265; doi:10.3390/nano14242055)
Supplement: Supplementary file 1 [file nanomaterials-14-02055-s001.zip › nanomaterials-3303215-supplementary.pdf]

Supplementary Information

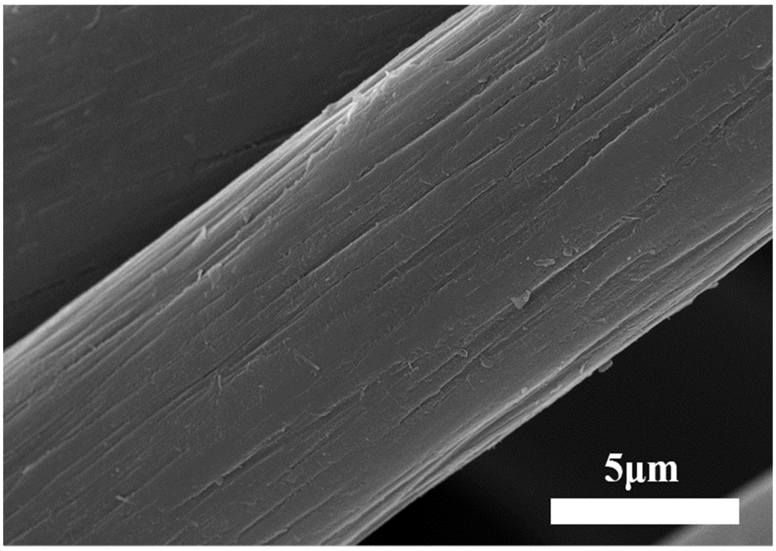

Figure S1. SEM image of P-GF.

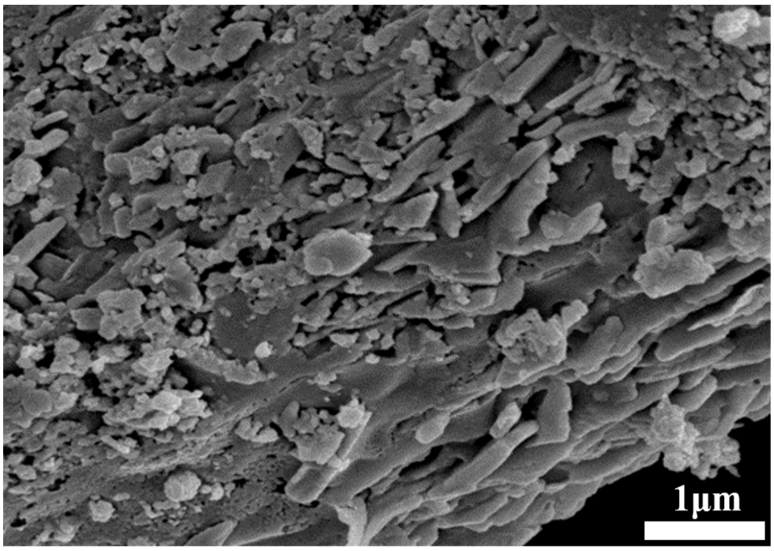

Figure S2. SEM image of Bi_2O_x-GF.

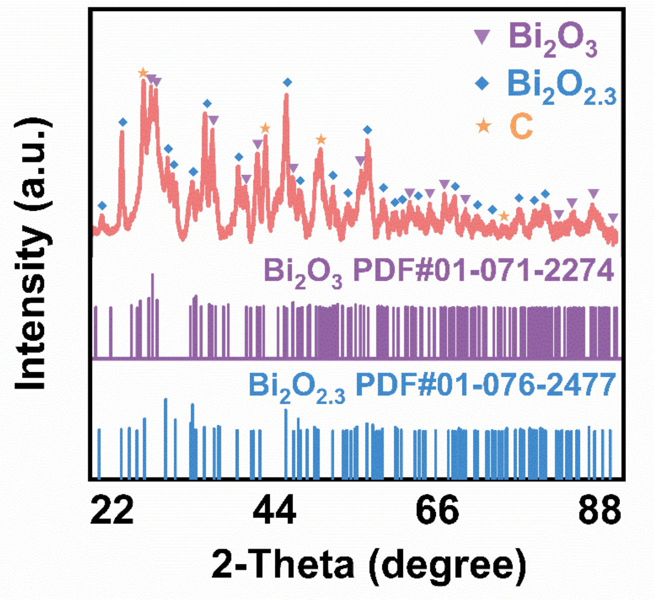

Figure S3. XRD pattern of the Bi_2O_x-GF

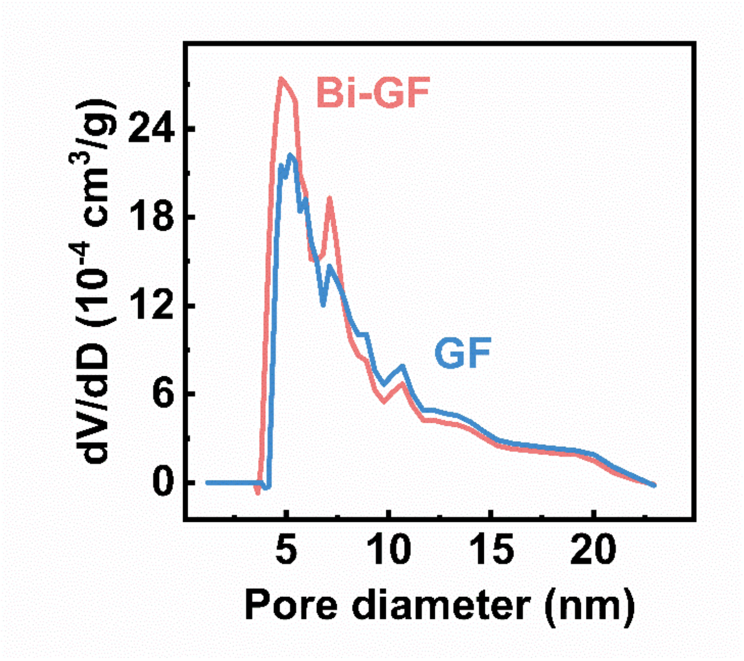

Figure S4. Pore size distribution of Bi-GF and GF electrodes

Table S1. BET specific surface areas of different electrodes.

electrode	Specific surface area (m^2/g)
GF	0.624
Bi-GF	5.862

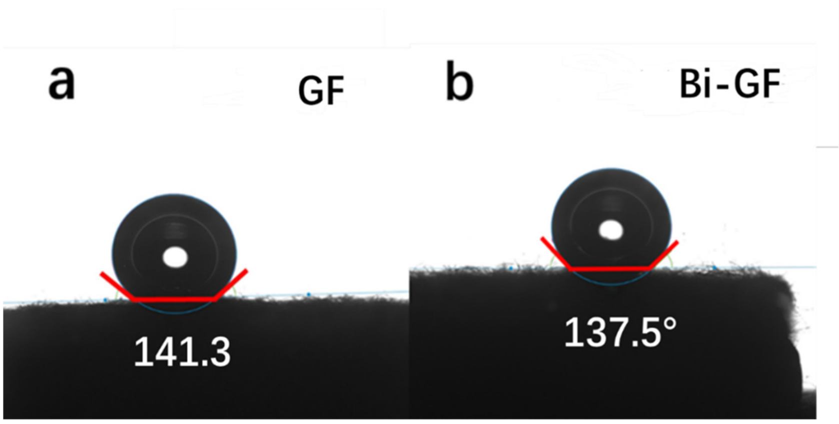

Figure S5. Water contact Angle test of (a) GF and (b) Bi-GF.

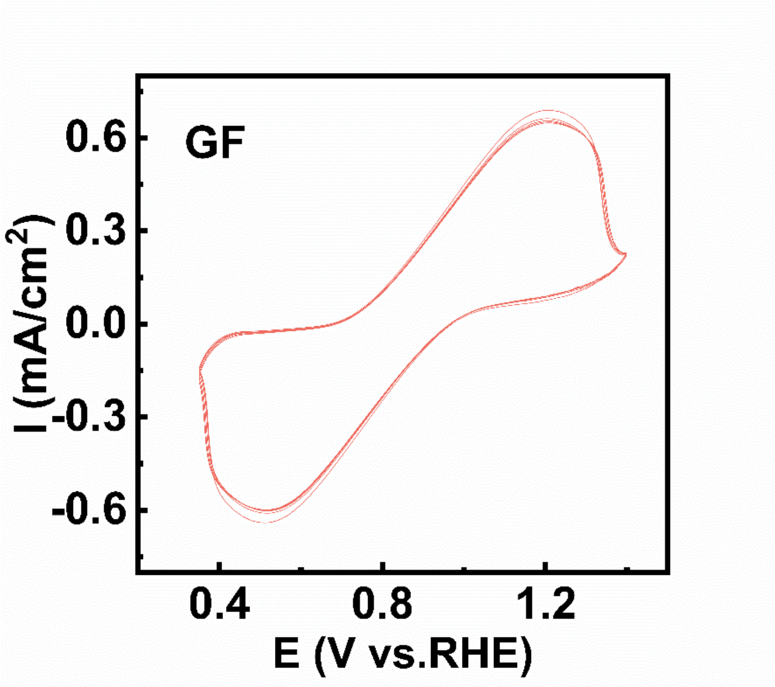

Figure S6. CV curves of the positive electrode of GF at 5 mV/s scanning rate.

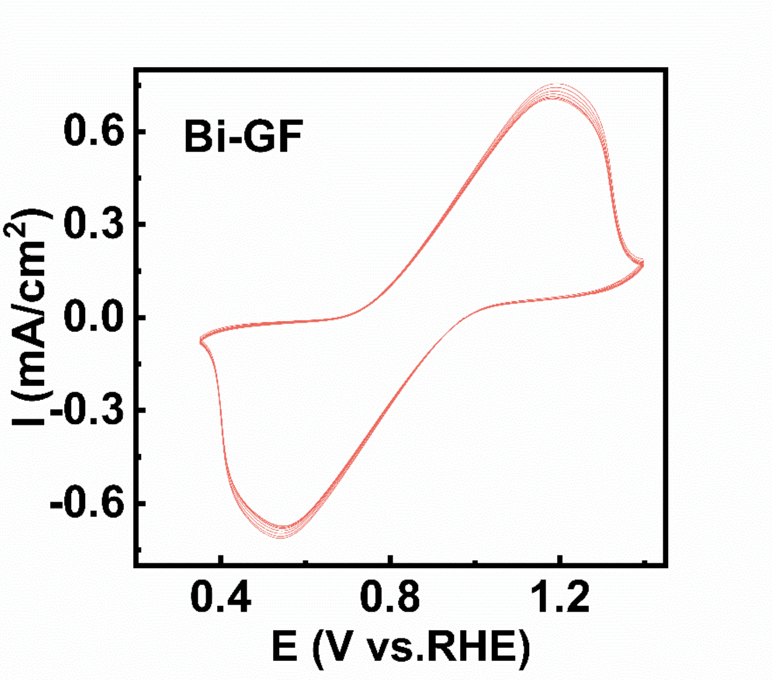

Figure S7. CV curves of the positive electrode of Bi-GF at 5 mV/s scanning rate.

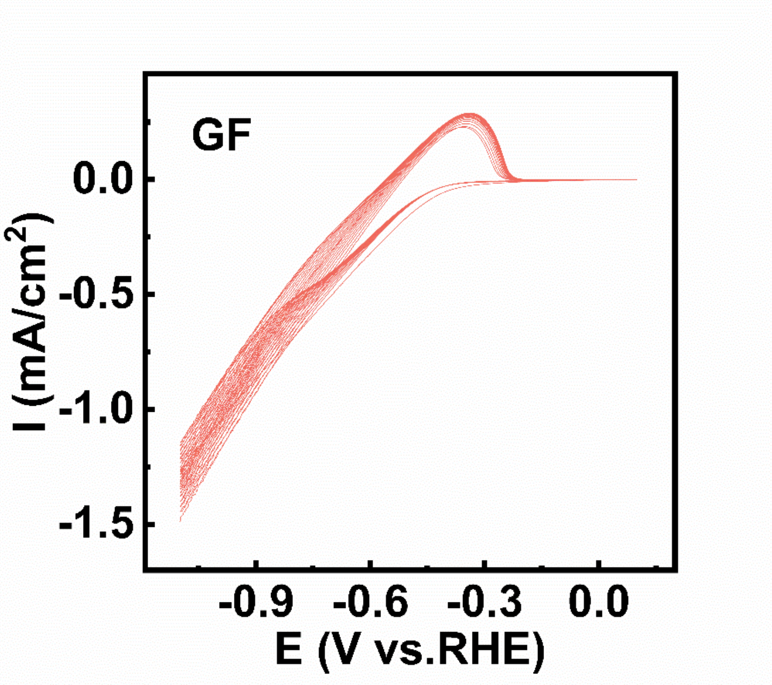

Figure S8. CV curves of the negative electrode of GF at 2 mV/s scanning rate.

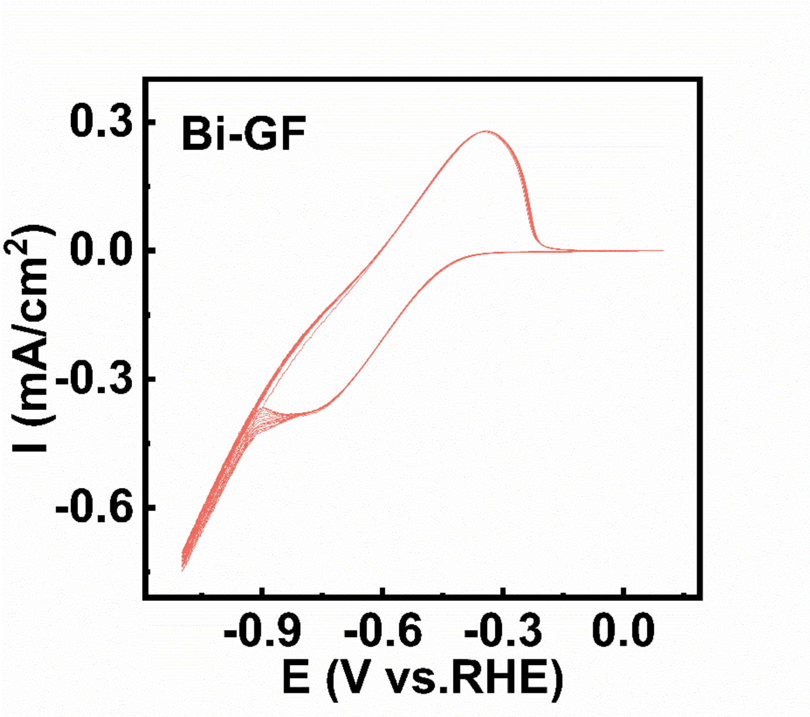

Figure S9. CV curves of the negative electrode of Bi-GF at 2 mV/s scanning rate.

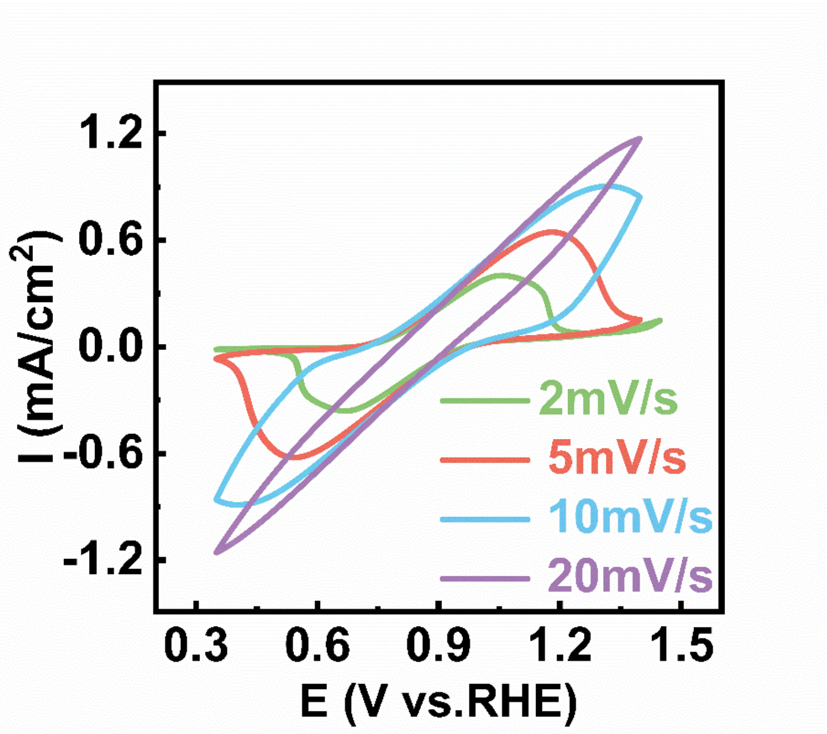

Figure S10. CV curves of the positive electrode of Bi-GF at different scanning rates.

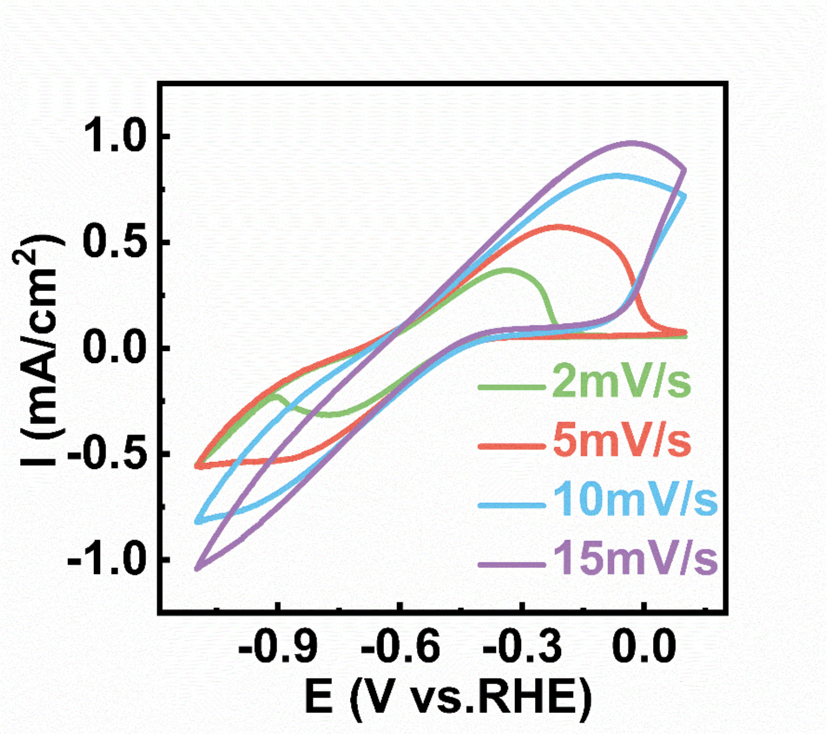

Figure S11. CV curves of the negative electrode of Bi-GF at different scanning rates.

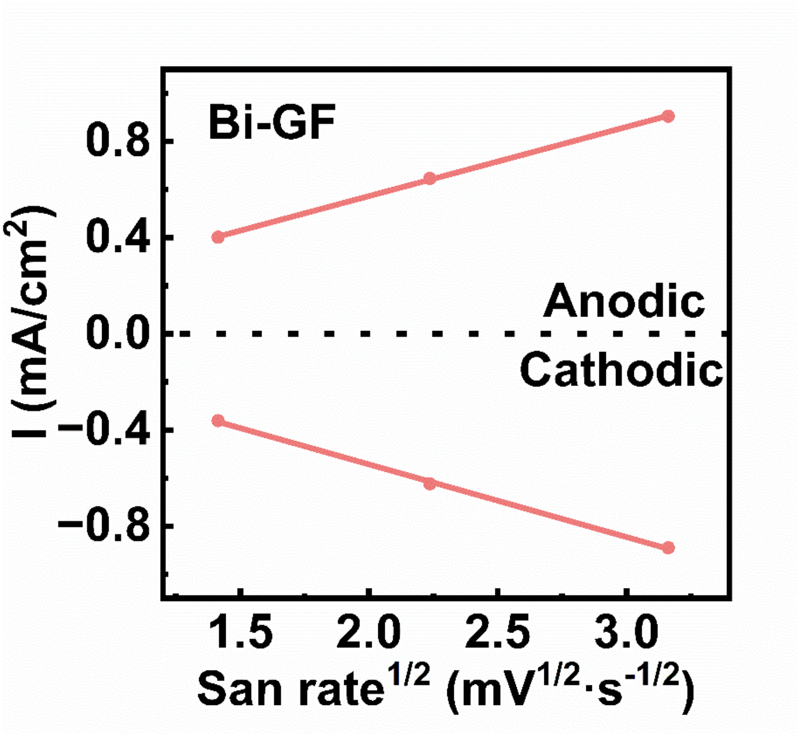

Figure S12. Relationship between peak current density of positive electrode and the square root of scan rate.

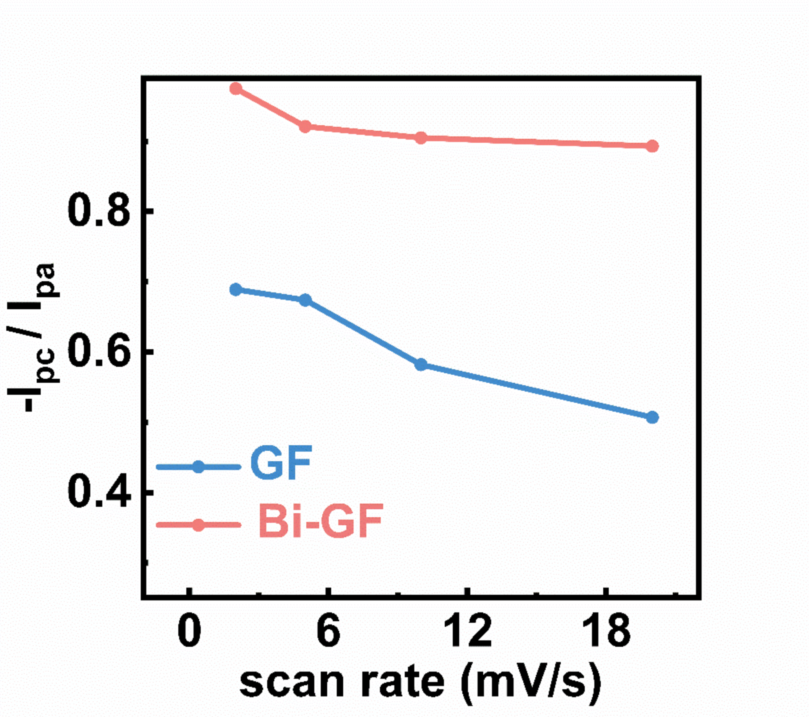

Figure S13. Redox peak current ratio of different electrodes at different scanning rates.

Table S2. Electrochemically available surface area of different electrodes.

electrode	EASA (cm^2)
GF	0.234
Bi-GF	0.249

Table S3. Impedance parameters of different electrodes at positive side.

electrode	R_s (mΩ)	R_{ct} (mΩ)
GF	330.6	45.0
Bi-GF	309.8	19.2

Table S4. Impedance parameters of different electrodes at negative side.

electrode	R_s (mΩ)	R_{ct} (Ω)
GF	507.0	32.8
Bi-GF	461.4	31.9

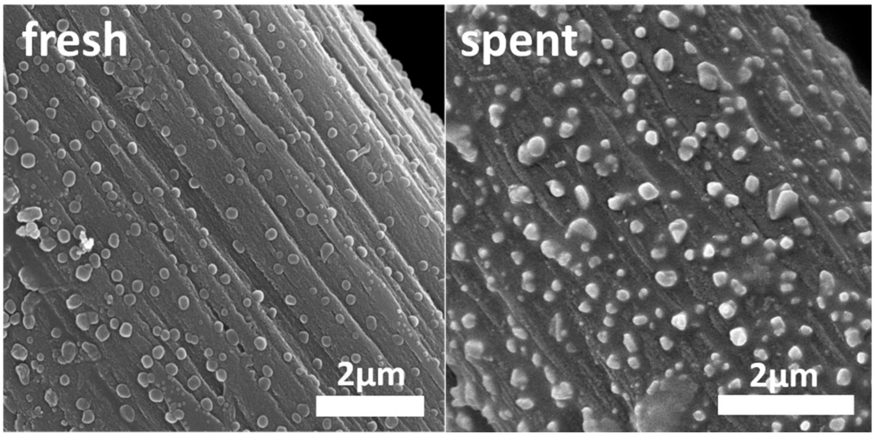

Figure S14. SEM of the Bi-GF electrode before and after 100 cycles of charge and discharge at a current density of 200 mA/cm^2.

Table S5. Comparison of battery performance at a current density of 200 mA/cm^2.

electrode material	energy efficiency	ref.
Bi@TGF	81.5%	[S 1]
Bi@GF	82.5%	[S 2]
GF500°C/$Ti_3C_2T_x$/Bi	80.1%	[S 3]
Bi NPs/NC@GF	82.99%	[S 4]
Bi-CN-GF/CN-GF	86.08%	[S 5]
**Bi-GF**	**83.05%**	

S 1. Liu, X.; Nie, Y.; Yu, L.; Liu, L.; Xi, J. Fine and dense bismuth electrocatalysts achieving high power density and cycling stability in vanadium flow batteries. *Journal of Energy Storage* **2024**, *91*, 112035, doi:https://doi.org/10.1016/j.est.2024.112035.

S 2. Ren, J.; Wang, Z.; Sun, J.; Guo, Z.; Liu, B.; Fan, X.; Zhao, T. In-situ electrodeposition of homogeneous and dense bismuth nanoparticles onto scale-up graphite felt anodes for vanadium redox flow batteries. *Journal of Power Sources* **2023**, *586*, 233655, doi:https://doi.org/10.1016/j.jpowsour.2023.233655.

S 3. Li, Q.; Pei, D.; Zhang, X.; Sun, H. Boosting performance of Ti3C2TX/Bi modified graphite felt electrode for all-vanadium redox flow battery. *Electrochimica Acta* **2024**, *473*, 143439, doi:https://doi.org/10.1016/j.electacta.2023.143439.

S 4. Fei, X.; Qiang, F.; Feng, X.; Jian, Z.; Haoyang, L.; Tao, L.; Xianfeng, L. Bismuth Single Atoms Regulated Graphite Felt Electrode Boosting High Power Density Vanadium Flow Batteries. *Journal of the American Chemical Society* **2024**, *146*, 26024-26033, doi:10.1021/jacs.4c04951.

S 5. Zhang, X.; Ye, X.; Huang, S.; Zhou, X. Promoting Pore-Level Mass Transport/Reaction in Flow Batteries: Bi Nanodot/Vertically Standing Carbon Nanosheet Composites on Carbon Fibers. *ACS Applied Materials & Interfaces* **2021**, *13*, 37111-37122, doi:10.1021/acsami.1c08494.
